# Photodynamic Therapy With YAG-OPO Laser for Early Stage Lung Cancer

**DOI:** 10.1155/DTE.4.75

**Published:** 1997

**Authors:** Harubumi Kato, Tetsuya Okunaka, Chimori Konaka, Kiyoyuki Furuse, Yoko Kusunoki, Takeshi Horai, Nobuhide Takifuji, Syunichi Negoro, Masahiro Fukuoka, Takashi Yaya, Ichiro Kawase

**Affiliations:** 1 Department of Surgery Tokyo Medical College Nishishinjuku Shinjuku-ku Tokyo 160 Japan; 2 Department of Internal Medicine National Kinki Central Hospital The Center for Adult Disease Osaka Japan; 3 Department of Field Research The Center for Adult Disease Osaka Japan; 4 4th Department of Internal Medicine The Center for Adult Disease Osaka Japan; 5 Department of Pulmonary Medicine Osaka City General Hospital Osaka Japan; 6 2nd Department of Internal Medicine Osaka Prefectural Habikino Hospital Osaka Japan

## Abstract

Photodynamic therapy (PDT) utilizing Photofrin is proving to be effective for the treatment of early stage lung cancers. The effect of PDT utilizing YAG-OPO laser as new light source was evaluated in 26 patients (29 lesions) with early stage lung cancers. YAG-OPO laser is solid state tunable laser which is easy to change wavelength between 620 and 670 nm exciting various kinds of photosensitizers. Moreover, YAG-OPO laser is more reliable, smaller and has less consumables than argon-dye laser or excimer-dye laser. As the result of PDT with YAG-OPO laser, complete remission (CR) was obtained in 82.6% of the 29 lesions, partial remission (PR) in 13.8% and no change (NC) was obtained in 3.4%. We conclude that PDT utilizing YAG-OPO laser is efficacious in the treatment of early stage lung cancers and can achieve complete remission.


*Diagnostic and Therapeutic Endoscopy*, Vol. 4, pp. 75–81
Reprints available directly from the publisher
Photocopying permitted by license only

© 1997 OPA (Overseas Publishers Association)
Amsterdam B.V. Published under license
in The Netherlands under the Harwood Academic
Publishers imprint, part of The Gordon and
Breach Publishing Group.
Printed in Singapore.

### Photodynamic Therapy with YAG-OPO Laser for Early Stage Lung Cancer

HARUBUMI KATO[a,*], TETSUYA OKUNAKA[a], CHIMORI KONAKA[a], KIYOYUKI FURUSE[b], YOKO KUSUNOKI[c], TAKESHI HORAI[d], NOBUHIDE TAKIFUJI[e], SYUNICHI NEGORO[e], MASAHIRO FUKUOKA[e], TAKASHI YAYA[f] and ICHIRO KAWASE[f]

[a] *Department of Surgery, Tokyo Medical College, Nishishinjuku, Shinjuku-ku, Tokyo 160, Japan;* [b] *Department of Internal Medicine, National Kinki Central Hospital;* [c] *Department of Field Research;* [d] *4th Department of Internal Medicine, The Center for Adult Disease, Osaka, Japan;* [e] *Department of Pulmonary Medicine, Osaka City General Hospital, Osaka, Japan;* [f] *2nd Department of Internal Medicine, Osaka Prefectural Habikino Hospital, Osaka, Japan*

*(Received 13 March 1997; In final form 30 May 1997)*

**Photodynamic therapy (PDT) utilizing Photofrin is proving to be effective for the treatment of early stage lung cancers. The effect of PDT utilizing YAG-OPO laser as new light source was evaluated in 26 patients (29 lesions) with early stage lung cancers. YAG-OPO laser is solid state tunable laser which is easy to change wavelength between 620 and 670 nm exciting various kinds of photosensitizers. Moreover, YAG-OPO laser is more reliable, smaller and has less consumables than argon-dye laser or excimer-dye laser. As the result of PDT with YAG-OPO laser, complete remission (CR) was obtained in 82.6% of the 29 lesions, partial remission (PR) in 13.8% and no change (NC) was obtained in 3.4%. We conclude that PDT utilizing YAG-OPO laser is efficacious in the treatment of early stage lung cancers and can achieve complete remission.**

*Keywords:* Photodynamic therapy, YAG-OPO laser, Photofrin, Early stage lung cancer

### INTRODUCTION

Increasing numbers of early stage lung cancer cases are being detected as a result of improved survey and diagnostic techniques. Despite the possibility of curative resection in many such cases, many of the patients are frequently at high surgical risk because of coexisting chronic obstructive pulmonary disease or cardiovascular disease. Considering the quality of life of the patients, it is essential to preserve lung tissue by treating the initial early stage lung cancer as conservatively as possible. The introduction of photodynamic therapy (PDT) has provided a new therapeutic alternative to surgery. PDT is a new cancer treatment modality that selectively destroys cancer cells by an interaction between absorbed light and a retained photosensitizer [1,2].

Since PDT with Photofrin and excimer-dye laser received approval from the Ministry of Health

* Corresponding author.

and Welfare of Japan in October 1994, increasing attention has been focused on this new treatment technique. Moreover, new photosensitizers of the second and the third generation have been developed, and tunable lasers are expected to apply for new generation photosensitizer [3].

This paper evaluates the YAG-OPO laser as a new light source for PDT in early stage lung cancer.

### PATIENTS AND METHODS

### Patient Eligibility

Eligibility criteria were as follows: (1) histologically and cytologically proven early stage lung cancer; no metastasis in hilar or mediastinal lymph nodes and no distant metastasis (stage 0: TisN0M0 or stage I: T1N0M0); (2) performance status (PS) of 0 to 2; (3) arterial oxygen pressure tension ($PaO_2$) greater than 60 Torr; (4) no tumor shadow on chest X-ray; (5) endoscopically visible distal tumor margins and accessibility to laser irradiation; (6) no previous treatment. Informed consent was obtained from all patients or their relatives.

### Laser Equipment

The light source employed in this trial was the YAG-OPO laser from Ishikawajima-Harima Heavy Industries Co., Ltd. (IHI, Tokyo, Japan). The YAG-OPO laser stands for an Optical Parametric Oscillator pumped by a *Q*-switched Nd:YAG laser. The wavelength of Nd:YAG laser (1,064 nm) is converted into a third (355 nm) by two nonlinear crystals. The THG of Nd:YAG laser (Third Harmonic Generation; 355 nm light: $\lambda_p$) pumps the OPO, which is composed of a resonator and a nonlinear crystal, as a result two different beams are generated by nonlinear effects. One beam is called the signal light ($\lambda_s$), and the other is the idler ($\lambda_i$). Generally, $\lambda_s$ is shorter than $\lambda_i$ and $\lambda_s$ is used as the irradiating beam. The combination of $\lambda_s$ and $\lambda_i$ can be changed easily, according to the angle of the OPO crystal. (Figs. 1 and 2). This system consists of a laser system, an operating stand, a chiller and an optical fiber. The laser emits a high peak power light beam between 620 and 670 nm. The beam can be irradiated onto tumors by two types of optical fibers.

The specifications are as follows:

(1) wavelength: 620–670 nm
(2) repetition rate: 25, 50 Hz
(3) Max. pulse energy: 6 mJ/pulse (output of optical fiber)
(4) Max. average power: 300 mW (6 mJ × 50 Hz)
(5) pulse width: 5–8 ns
(6) peak power: $< 1$ MW

When the parameters are set at the operating stand, the YAG-OPO laser system automatically starts up and stands by. The parameters displayed on the color LCD touched panel are repetition rate, wavelength, pulse energy, total energy, type of fiber tip (forward-expanded irradiating tip and side irradiating tip) and remaining time. The repetition rate, wavelength, pulse energy, total energy and type of fiber tip can be changed easily on the control panel. For reliability, the computer controlling system stabilizes power and wavelength and fiber calibration. Furthermore, the function of photo-coagulation for YAG laser (Max. Power: 10 W) is available as an option [4].

### Procedure

Bronchoscopical PDT is performed with topical anesthesia approximately 48 h after the intravenous injection of 2.0 mg/kg body weight of Photofrin. After the injection of Photofrin, the patients are instructed to avoid direct sunlight for at least four weeks. The YAG-OPO laser emits a 630 nm wavelength beam which has the deepest tissue penetration among the wavelengths that excite Photofrin. The laser beam is transmitted via a quartz fiber (400 mm) inserted through the instrumentation channel of a fiberoptic bronchoscope.

The pulse energy of the output at the fiber tip was adjusted to 4 mJ/pulse. The frequency was 50 Hz, giving energy densities of 100 J/cm$^2$. After

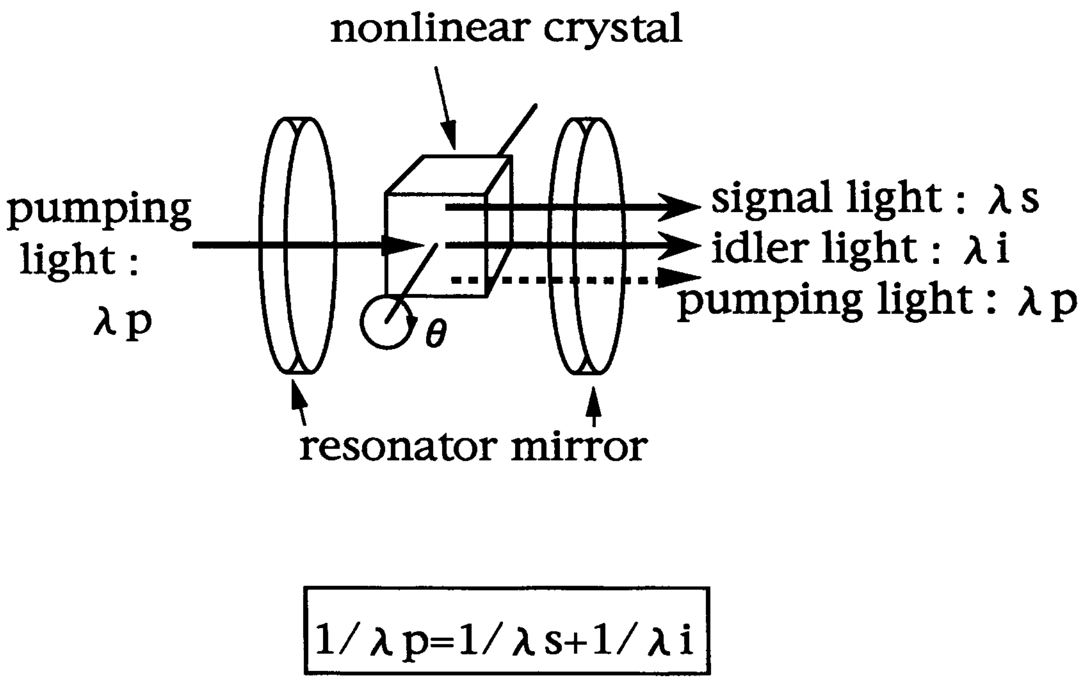

FIGURE 1 The principle of an optical parametric oscillation. $\lambda_p$, $\lambda_s$, and $\lambda_i$ are wavelength of each lights.

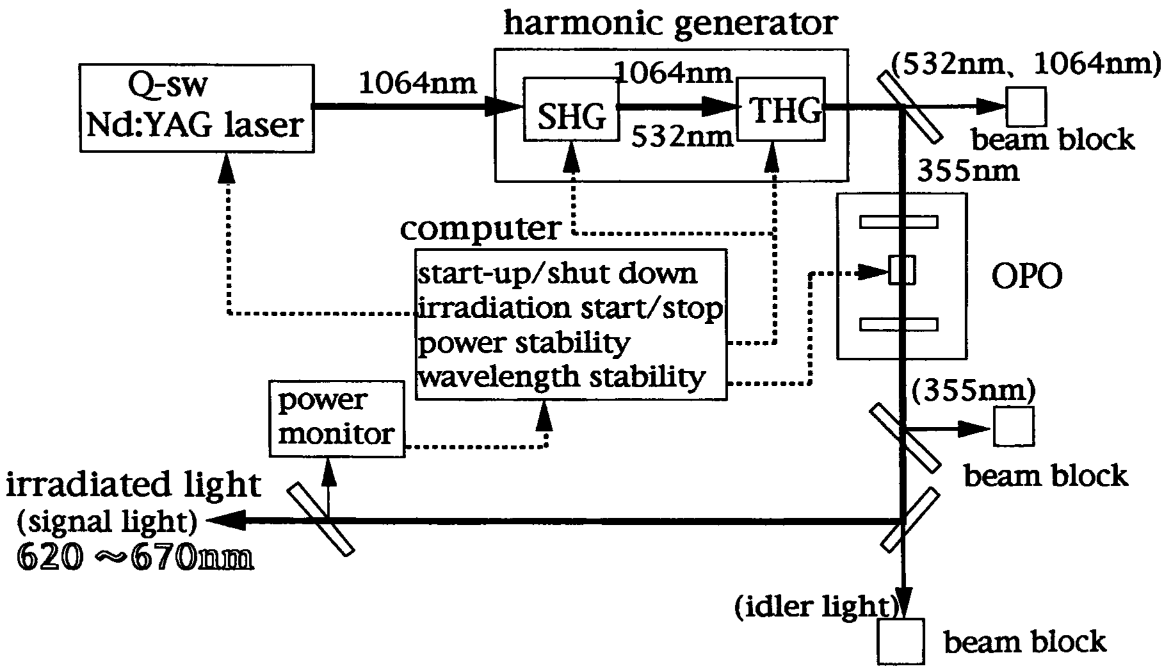

FIGURE 2 The function of YAG-OPO laser system. A computer controls the total laser system, and the stable laser beam is emitted.

the PDT procedure, bronchial toilet was performed every 2 or 3 days for 1 week.

Tumor response was classified into three grades: complete remission (CR), when no tumor was observable by biopsy and/or brushing cytology for at least 4 weeks; partial remission (PR) which is defined as a reduction in tumor volume greater than 50% but with the cancer still recognizable on biopsy or brushing for at least 4 weeks after therapy; and no change (NC) which is defined as no change in tumor size or a decrease of less than 50% and the cancer still recognizable by biopsy or brushing. One month after treatment, the tumor response to PDT was evaluated endoscopically, roentgenographically, cytologically, and histologically.

### RESULTS

### Patient Characteristics

From May 1995 through December 1996, 28 patients with 31 carcinomas were entered into this study. However 2 patients with 2 carcinomas were excluded, because of lesions lacking clear visibility of the distal tumor margins. Twenty-six male patients with 29 carcinomas were eligible for response and side effect assessment. Characteristics of the eligible patients are listed in Table I. The age distribution ranges from 53 to 79 years old. According to tumor staging, 10 carcinomas were identified as stage 0 (TisN0M0) and 19 as stage I (T1N0M0). Histologically, all cases were squamous cell carcinoma. Twenty-two carcinomas had a longitudinal tumor extent of smaller than 1 cm and 7 carcinomas had a longitudinal tumor extent of greater than 1 cm.

TABLE I Patients characteristics

| | |
|---|---|
| No. of patients | 26 |
| Age (years) | |
| Median | 69.0 |
| Range | 53–79 |
| Sex (male/female) | 26/0 |
| PS | |
| 0 | 19 |
| 1 | 7 |
| No. of carcinoma | 29 |
| Tumor stage | |
| 0 (TisN0M0) | 10 |
| I (T1N0M0) | 19 |
| Histlogy, squamous cell | 29 |
| Bronchoscopic appearance | |
| Superficial type | 16 |
| Nodular | 13 |
| Tumor size | |
| $<0.5$ | 10 |
| 0.5–0.9 | 12 |
| 0.9–1.9 | 6 |
| $2.0\leq$ | 1 |

### Response

Tumor response to PDT in 29 carcinomas is shown in Table II. Table III shows the summary of results of PDT. CR was obtained in 24 lesions out of 29 lesions (82.6%), PR in 4 lesions and NC in one lesion. According to tumor stage, of the 10 carcinomas identified as stage 0 (TisN0M0), CR was obtained on 9 (90%) carcinomas and PR in 1. Of the 19 carcinomas identified as stage I (T1N0M0), 15 (78.9%) carcinomas have CR, 3 carcinomas have PR and 1 carcinoma have NC. Of the 9 CR cases, 2 recurred locally and were then treated by PDT again, and CR was achieved. One patient died with heart failure.

The therapeutic effectiveness of PDT was analyzed according to the longitudinal tumor size. The univariate analysis was based on $2 \times 2$ tables and differences were tested by the $\chi^2$ test. Of the 22 cancer lesions that had a longitudinal tumor extent of 1 cm or less, 19 (86.4%) obtained a CR after initial PDT, however of the 7 carcinomas that had a longitudinal tumor extent of greater that 1 cm, 6 (85.7%) showed CR after PDT. There was no significant difference between the two groups.

### Adverse Effect

There were no adverse effects related to the YAG-OPO laser irradiation, although mild skin photosensitivity caused by Photofrin was seen in 4 patients.

TABLE II Response of PDT with YAG-OPO laser

| No. | Age | Sex | Location | Tumor size mm × mm | Histologic type | Clinical stage | Bronchoscopic appearance | Response | Recurrence |
|---|---|---|---|---|---|---|---|---|---|
| 1 | 79 | M | bif*. of left upper & lower bronchi | 3 × 3 | Sq* | I | nodular | CR | – |
| 2 | 56 | M | left $B^3$ | 2 × 3 | Sq | I | superficial | CR | – |
| 3 | 68 | M | right $B^2$ | 15 × 15 | Sq | I | nodular | CR | + |
| | | | left $B^{1+2}$ | 3 × 3 | Sq | I | nodular | CR | – |
| 4 | 67 | M | right $B^3$–$B^{1.2}$ | 5 × 3 | Sq | 0 | superficial | CR | – |
| 5 | 79 | M | right $B^{1+2}$ | 15 × 15 | Sq | I | nodular | CR | + |
| 6 | 72 | M | right $B^1$–$B^3$ | 5 × 5 | Sq | I | superficial | CR | – |
| 7 | 70 | M | right $B^3$ | 1 × 1 | Sq | I | superficial | CR | – |
| 8 | 71 | M | trachea | 2 × 3 | Sq | I | nodular | CR | – |
| 9 | 71 | M | right $B^{10}$ | 5 × 5 | Sq | I | superficial | PR | |
| 10 | 71 | M | left $B^{1+2}$ | 5 × 5 | Sq | 0 | superficial | PR | |
| 11 | 65 | M | bif. of left upper & linguar bronchi ~ orifice of left $B^3$ | 15.7 × 20 | Sq | 0 | superficial | CR | – |
| 12 | 74 | M | left $B^3$ | 5 × 3 | Sq | I | superficial | CR | – |
| 13 | 71 | M | right $B^{10}$ | 4 × 3 | Sq | I | nodular | CR | – |
| 14 | 75 | M | left $B^6$ | 7 × 3 | Sq | I | superficial | CR | – |
| 15 | 66 | M | left $B^3$ | 5 × 4 | Sq | 0 | nodular | CR | – |
| 16 | 67 | M | right $B^{1+3-2}$ | 10 × 7 | Sq | 0 | superficial | CR | – |
| 17 | 65 | M | left $B^3_a$ | 3 × 3 | Sq | I | nodular | CR | – |
| 18 | 76 | M | right $B^3_b$ | 3 × 3 | Sq | 0 | superficial | CR | – |
| 19 | 70 | M | bif. of left upper & lower bronchi | 3 × 3 | Sq | I | superficial | CR | – |
| 20 | 65 | M | orifice of left upper lobe bronchi | 3 × 3 | Sq | 0 | superficial | CR | – |
| | | | orifice of right upper lobe bronchi | 7 × 7 | Sq | 0 | superficial | CR | – |
| 21 | 72 | M | trachea | 10 × 10 | Sq | I | nodular | PR | |
| 22 | 72 | M | left $B^3$ | 3 × 6 | Sq | I | nodular | PR | |
| 23 | 53 | M | left $B^{1+2}$ | 3 × 5 | Sq | I | nodular | NC | |
| | | | left $B^{10}$ | 12 × 5 | Sq | I | nodular | CR | – |
| 24 | 66 | M | left $B^3$–$B^4$ | 5 × 4 | Sq | I | superficial | CR | – |
| 25 | 64 | M | left $B^8$ | 10 × 6 | Sq | 0 | superficial | CR | – |
| 26 | 68 | M | left $B^6$ | 5 × 5 | Sq | 0 | nodular | CR | – |

Sq*: squamous cell carcinoma; bif*: bifurcation.

### Case Report

The case no. 5, 79-year-old man, squamous cell carcinoma of the lung was initially diagnosed based on positive sputum cytology. The tumors were nodular, located in the right $B^{1+2}$. The size of the tumor is 15 mm by 15 mm in size. Since the patient's pulmonary function was very poor, he was subsequently treated by PDT. Figure 3 shows the tumor in the right $B^{1+2}$, before and 2 months after PDT. He is now apparently disease-free 17 months after PDT.

### DISCUSSION

Photodynamic therapy, a relatively new modality used in the treatment of cancer, has gained considerable acceptance in the past decade. A wide variety of malignancies have been treated by this method and according to the literature, over 3000 patients worldwide have been treated with photodynamic therapy [5]. The estimate of the number of institutions and investigators involved with PDT worldwide is about 90 and 180, respectively. In Japan, PDT with Photofrin and excimer dye

TABLE III Summary of response

| | No. of Carcinomas | Response | | | Recurrence |
|---|---|---|---|---|---|
| | | CR | PR | NC | |
| Overall | 29 | 24 (82.6%) | 4 (13.8%) | 1 (3.4%) | 2 (6.9%) |
| Tumor stage | | | | | |
| 0 (TisN0M0) | 10 | 9 (90%) | 1 | 0 | 0 |
| I (T1N0M0) | 19 | 15 (78.9%) | 3 | 1 | 2 |
| Bronchoscopic appearance | | | | | |
| Superficial type | 16 | 15 (93.8%) | 1 | 0 | 1 |
| Nodular | 13 | 10 (76.9%) | 2 | 1 | 2 |
| Tumor size | | | | | |
| <0.5 | 10 | 10 (100%) | 0 | 0 | 0 |
| 0.5–0.9 | 12 | 9 (75.0%) | 2 | 1 | 1 |
| 0.9–1.9 | 6 | 5 (83.3%) | 1 | 0 | 2 |
| 2.0≤ | 1 | 1 (100%) | 0 | 0 | 0 |

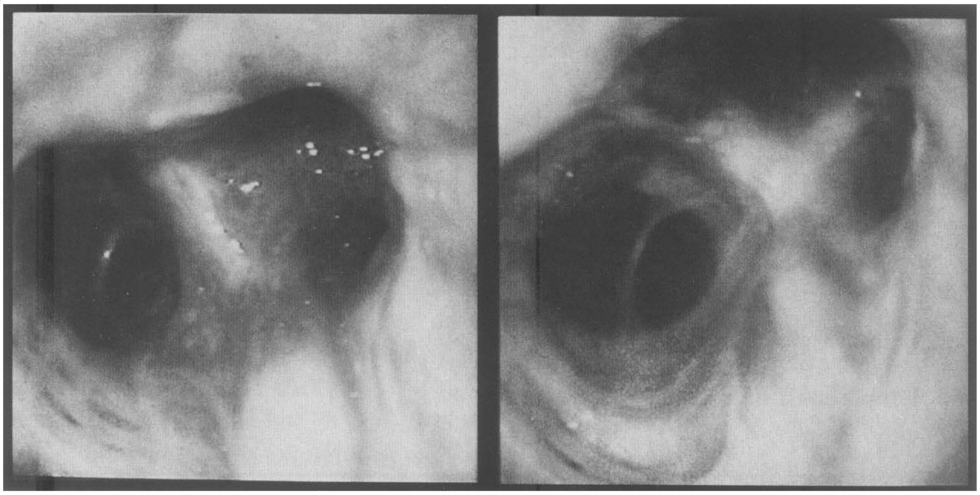

a b

FIGURE 3 Endoscopical findings of case no. 5, 79-year-old man, squamous cell carcinoma of the lung. The tumors were nodular, located in the right $B^{1+2}$. The size of the tumor 15 × 15 mm in size (left) and bronchoscopic finding of same site 2 months after PDT.

laser obtained government approval in October 1994 and finally obtained national insurance reimbursement status in April 1996 [3].

As the cost of medical care is one of the important problems, we evaluated the cost-effectiveness of PDT in early stage lung cancer cases against lobectomy. Effectiveness was determined using quality adjusted life years saved (QALYs) which is the 5-year survival rate adjusted in terms of the quality of life of the patient, and cost-effectiveness rate was obtained based on the costs of treatment methods during patient's stay in the hospital. Health care costs, including drugs, were calculated according to the 1993 National Health Insurance list. The total cost of the operated group is $14,948 and that for the PDT group is $8,475.

The cost-effectiveness rate of the operated group, that is the average cost of treatment per post-operative living month, was $313, while that of the entire PDT group was $250. This indicates that the cost-effectiveness rate for the operated group was apparently 1.3 times higher than that of the PDT group. The monthly cost-effectiveness rate for the PDT group of superficial lesions smaller than 2 cm was $213 in which the cost was cheaper in the PDT group [6].

The excimer-dye laser consists of a gas and dye laser, which means that the system is large and the maintenance cost is high. Therefore, a small solid state laser would be useful for PDT. Since the YAG-OPO laser is all solid state laser except for the flash lamps, it is more reliable, smaller and has less consumables than argon-dye laser or excimer-dye laser [4]. Moreover, the tunability of its wavelength enables it to be used with various kinds of photosensitizers; hematoporphyrin derivative (630 nm), 5-ALA (630 nm), pheophorbide-a (650 nm), mono-L-aspartyl chlorin e6 (664 nm), aluminum phthalocyanine (670 nm), etc [7].

In a multi-center research study in Japan on early stage lung cancer treated by PDT with Photfrin and excimer-dye laser involving 66 carcinomas, CR was achieved in 51 carcinomas (77.3%) after the initial PDT [8,9]. Our results with the YAG-OPO laser were by no means inferior to those data and also there was no serious adverse effect by the laser itself except slight skin photosensitivity which was caused by Photofrin. These data encourage the future clinical use of the YAG-OPO laser.

We previously reported that complete remission was difficult to obtain in lesions which were anatomically difficult to photoirradiate. However, the side irradiating tip made it possible to photoradiate from an angle of 90° to the surface of the lesion in this study.

PDT holds great future potential in the curative treatment of early stage cancer, palliative treatment of advanced cancer for local improvement of lesions, combination therapy with surgery and with ionizing radiation and chemotherapy. QOL can be maintained by PDT especially in the elderly and patients with multiple lung cancers. PDT is cost-effective in comparison with other treatments. When new photosensitizers which distribute more equally in the tumor tissue which yields deeper tissue penetration due to its longer wavelength are used clinically, more successful results will be obtained with YAG-OPO laser.

Recent studies on photodynamic therapy started just 25 years ago. Therefore there are still a number of unsolved problems. However it is clear that PDT is one of the new therapeutic strategies for early stage central type lung cancer.

### *Acknowledgements*

We thank Professor J.P. Barron for his review of the manuscript and Mr. Keigo Takaoka and Mr. Takeshi Udagawa for their help to collect data. This work was supported by Japan Science and Technology Corporation.

### *References*

[1] Dougherty, T.J., Laurence, G. Kaufman, J.H. *et al.* A. Photoradiation in the treatment of recurrent breast carcinoma. *J. Natl. Cancer Inst.* 1979; **62**: 231–237.

[2] Hayata, Y., Kato, H. Konaka, C. *et al.* Hematoporphyrin derivative and laser photoradiation in the treatment of lung cancer. *Chest* 1982; **81**: 269–277.

[3] Kato, H., Okunaka, T. and Shimatani, H. Photodynamic therapy for early stage bronchogenic carcinoma. *J. Clin. Laser Med. Surg.* 1996; **5**: 235–238.

[4] Udagawa, T., Inoue, K., Fukutomi, S. and Takaoka, K. YAG-OPO Laser for PDT Application. *J. Jpn. Society for Laser Med.* 1995; **16**(4): 25–30.

[5] Marcus, S.L. and Dugan, M. Global status of clinical photodynamic therapy: The registration process for a new therapy. *Lasers Surg. Med.* 1992; **12**: 318–324.

[6] Fujino, S., Ogawa, K., Kato, H. and Okunaka, T. Cost effectiveness of PDT for early stage lung cancer (Japanese). *Shinryou to Shinyaku* 1994; **31**: 2101–2127.

[7] Kato, H. and Okunaka, T. Photodynamic therapy in early tumors. In *Minimally Invasive Techniques in Thoracic Medicine and Surgery* (M. Hetzel, Ed.), Chapman and Hall Medical, London, 1995; 149–172.

[8] Furuse, K., Fukuoka, M., Kato, H. *et al.* A prospective phase II study on photodynamic therapy with Photofrin II for centrally located early stage lung cancer. *J. Clin. Oncol.* 1993; **11**: 1852–1857.

[9] Kato, H., Horai, T., Furuse, K. *et al.* Photodynamic therapy for cancers: A clinical trial of Porfimer sodium in Japan. *Jpn. J. Cancer Res.* 1993; **84**: 1209–1214.

